# Response of WUE of maize at ear stage to the coupling effect of CO_2_ and temperature

**DOI:** 10.1016/j.heliyon.2023.e23646

**Published:** 2023-12-19

**Authors:** Sicong Sun, Xinquan Hu, Yongsheng Wei, Xiaoxiao Chen, Yanzheng Li, Jun Cao

**Affiliations:** a College of Life Sciences, Northwest A & F University, Yangling, 712100, China; b College of Life Sciences, Zhejiang University, Hangzhou, 310058, China

**Keywords:** CO_2_ concentration, Maize, Response model, Temperature, WUE

## Abstract

In the face of global warming, the photosynthesis and transpiration of plants will change greatly, which will ultimately affect the water use efficiency (WUE) of plants. In order to study the coupling effects of CO_2_ and temperature on WUE of maize at ear stage, ‘Zhengdan 958’ was taken as the research object, and 5 temperatures (20 °C, 25 °C, 30 °C, 35 °C and 40 °C) and 11 CO_2_ concentration (400, 300, 200, 150, 100, 50, 400, 400, 600, 800 and 1000 μmol mol^−1^) were set to measure the parameters such as net photosynthetic rate (*P*_n_), transpiration rate (*T*_r_), stomatal conductance (*G*_s_) and intercellular CO_2_ concentration (*C*_i_) of single leaves. The response of WUE (*P*_n_/*T*_r_) to CO_2_ and temperature was evaluated by a CO_2_ response model. The results show that at the same temperature, *P*_n_ and WUE increased with CO_2_ level, while *T*_r_ decreased as CO_2_ level increases; at the same CO_2_ concentration, *P*_n_ and *T*_r_ were both positively correlated with temperature, while WUE decreased with the increase of temperature. The maximum value of WUE was obtained when the CO_2_ concentration was 1000 μmol mol^−1^ and the temperature was 20.0 °C. The results suggest that global warming will not improve WUE of maize, which will bring more severe challenges to water-saving agriculture and food security.

## 1 Introduction

Since 1750, with the rapid growth of the world population and the use of fossil fuels, the global atmospheric CO_2_ concentration has increased from 280 μmol mol^−1^ to about 400 μmol mol^−1^, and it is expected to reach 800 μmol mol^−1^ by the end of the 21st century [1]. However, the increase of atmospheric CO_2_ concentration will intensify the greenhouse effect. According to the data of IPCC AR6 [2], the global average temperature will increase by 1.5 °C ∼ 2 °C by the end of 21st century, which will directly affect physiological processes such as photosynthesis and transpiration of plants [3], and affect WUE.

WUE is usually defined as the amount of CO_2_ assimilation or dry matter production per unit mass of water consumed by plants [4], which is an important parameter to describe the water use characteristics of plant leaves and reflect the response of plants to climate change. Under the realistic conditions of insufficient global water resources, increasing population, and growing demand for food, improving WUE is of great significance for agricultural production, ecological maintenance and construction. However, in the existing research on WUE, temperature or CO_2_ is mostly studied as a standalone factor [5], while the research on the coupling effect of two factors on WUE is scarce [6]. In terms of research methods, by analyzing the CO_2_ response curves of *P*_n_, *T*_r_ and WUE respectively, important physiological parameters such as CO_2_ compensation point (CCP), maximum net photosynthetic rate (*A*_max_) and CO_2_ saturation point (CSP) can be obtained, and the understanding of crop WUE under global climate change can be improved.

Maize is an important food and feed crop, with great production potential and high economic efficiency; it has food, feed and various industrial uses, and is of strategic importance in guaranteeing food security. In the previous studies on maize, researchers have concluded that higher CO_2_ improved average WUE [7,8], and high temperatures have a linear negative effect on WUE [9]. Although the increase of CO_2_ concentration is beneficial to raise maize yield and WUE, it is hard to offset the negative effects of the increase in temperature. Therefore, with maize as the experimental material, this study quantitatively evaluated the effects of CO_2_ concentration and temperature on WUE by establishing CO_2_ response models of *P*_n_, *T*_r_ and WUE at different temperatures, so as to provide theoretical basis for the research and practice of WUE improvement on maize under the background of global warming.

## 2 Materials and methods

### 2.1 Plant material and growth conditions

The experimental site was located in the Northwest A&F University (34°17′N, 108°4′E) in Yangling District, Xianyang City, Shaanxi Province. *Zea may* L. ‘Zhengdan 958’, the main maize variety in Guanzhong area of Shaanxi Province, was used as the experimental material and cultivated in an open-air incubator (52 cm × 39 cm × 29.5 cm). The culture medium was local farmland topsoil, with 10 seedlings per box, and a total of 3 boxes, as 3 replicates. Sown on June 6, 2021, each box/seedling received 8 L of water every 7 days, and did not receive water when the rainfall is more than 5 mm in the week. The related indicators were measured from August 23 to 27 at 8: 30 to 11: 30 and from 14: 30 to 18: 00 every day, the relevant results are shown as an average of the five days' data.

### 2.2 Determination of gas exchange parameters

**Fig. 1:**
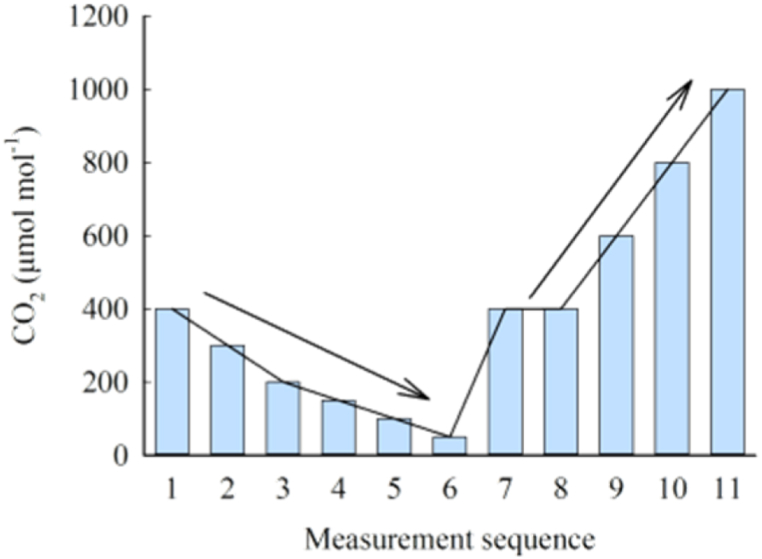
Schematic diagram of measurement sequence.

The photosynthetic-CO_2_ response curve of maize at heading stage was measured using LI-6400 (LI-COR Inc., USA), a portable photosynthetic apparatus. The parameters such as net photosynthetic rate (*P*_n_), transpiration rate (*T*_r_), stomatal conductance (*G*_s_) and intercellular CO_2_ concentration (*C*_i_) were obtained. Using the LI-6400 temperature control module on the basis of the ambient temperature, five temperature levels (20 °C, 25 °C, 30 °C, 35 °C and 40 °C) were obtained, and 11 CO_2_ concentration (400, 300, 200, 150, 100, 50, 400, 400, 600, 800 and 1000 μmol mol^−1^, Fig. 1) were set. The photosynthetically active radiation (PAR) in the leaf chamber was controlled to 1200 μmol m^−2^ s^−1^, and other environmental factors remained in the natural state. The first fully expanded leaf with consistent growth rate from top to bottom was measured in each plant to get *P*_n_, *T*_r_, *G*_s_ and *C*_i_, and each leaf was measured twice, and the average value represented one photosynthetic index. Before measurement, each plant was balanced for 10 min under the conditions of light intensity of 1200 μmol m^−2^ s^−1^, CO_2_ concentration of 400 μmol mol^−1^, temperature of (25 ± 0.2)°C and relative humidity of (71.2 ± 2.9)%. The WUE of leaves is defined as WUE = *P*_n_/*T*_r_.

In Fig. 1, the gray bar graph represents the applied CO_2_ concentration, and the arrowhead indicates the measuring direction.

### 2.3 Model building and data processing

#### 2.3.1 Response curve of *P*_n_ to CO_2_ at different temperatures

Because the right-angled hyperbola modified model has a high degree of fitting for the response curves of plants in the stages of activation, inhibition and adaptation, which can directly calculate CSP, CCP and *A*_max_ [10], this model was used to fit the CO_2_ response curves of *P*_n_ at different temperatures:

(1) $$P_{n}=a\frac{1-bC_{a}}{1+cC_{a}}C_{a}-d$$

where *P*_n_ is the net photosynthetic rate, *a* is the initial carboxylation efficiency of CO_2_ response curve, *C*_*a*_ is the atmospheric CO_2_ concentration, and *b*, *c* and *d* are the model evaluation coefficients (the units of *b* and *c* are mol μmol^−1^, and the unit of *d* is μmol m^−2^ s^−1^).

#### 2.3.2 Estimation of CSP, *A*_max_ and CCP

CSP was obtained by solving the first derivative of equation (1) and calculating the solution when its derivative value is 0 (Method 1, Analytical solution of the model).

(2) $$\text{CSP}=\frac{\sqrt{\left(b+c\right)/b}-1}{c}$$

*A*_max_ was obtained by substituting equation (2) into equation (1) (Method 1), as equation (3):

(3) $$A_{\max}=a{\left(\frac{\sqrt{b+c}-\sqrt{b}}{c}\right)}^{2}-d$$

CCP was obtained by substituting *P*_n_ = 0 into equation (1) (Method 1), as equation (4):

(4) $$\text{CCP}=\frac{a-cd-\sqrt{a^{2}-2ad\left(2b+c\right)+c^{2}d^{2}}}{2ab}$$

Linear fitting was made for *P*_n_ in the range of 600, 800 and 1000 μmol mol^−1^, and *P*_n_ in the range of 400 μmol mol^−1^–400 μmol mol^−1^ (1st ∼ 6th and 8th CO_2_ concentration) respectively:

(5) $$P_{n}=kC_{a}-d$$

**Fig. 2:**
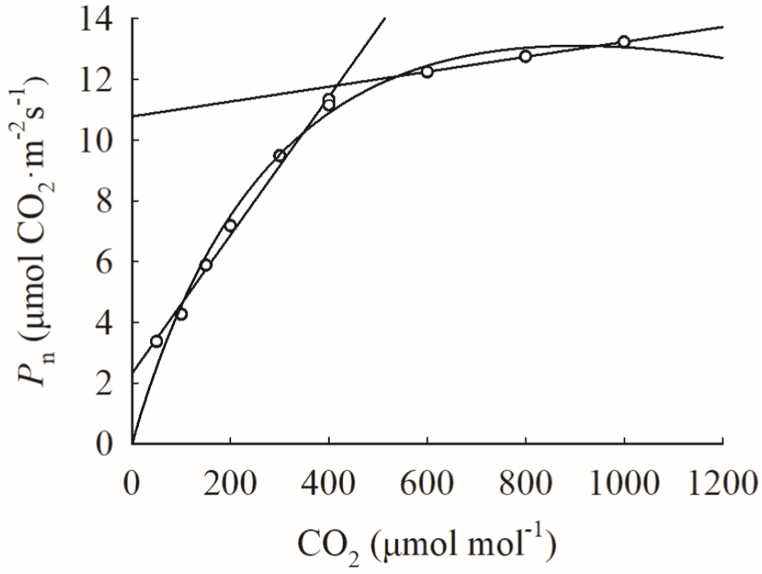
Schematic diagram of linear fitting method. Points are the average measurements of the photosynthetic CO_2_ response at any set of test temperatures, and two straight lines are fitted by data before 400 μmol mol^-1^ and data after 400 μmol mol^-1^ respectively. The intersection of the straight line fitted by data before 400 μmol mol^-1^ and the X axis is CCP; the abscissa of the intersection of two straight lines is the estimated value of CSP, and the ordinate is the estimated value of *A*_max_.

In equation (5), *k* is the parameter to be estimated, and other parameters keep the same meanings as in equation (1). The abscissa of the intersection of two straight lines is the estimated value of CSP, and the ordinate is the estimated value of *A*_max_. The intersection of the fitting curve of 400 μmol mol^−1^–400 μmol mol^−1^ and the positive semi-axis of X axis is the estimated value of CCP (Method 2, Linear fitting estimation, Fig. 2).

#### 2.3.3 Response curve of WUE to CO_2_ at different temperatures

In order to evaluate the adaptability of plants to elevated CO_2_, the CO_2_ concentration set up in this study is different from that in other CO_2_ response curves, which also led to the discontinuous response of WUE to CO_2_, great differences in the numerical values at the breakpoints, and a coefficient of determination (*R*^2^) for fitting the right-angled hyperbolic correction model approached zero. Therefore, the quadratic function piecewise fitting is carried out for WUE (the first stage: 0–400 μmol mol^−1^, and the second stage: 400–1000 μmol mol^−1^, and *T*_r_ is also different before and after the segmentation, so only the slope is calculated and the model fitting is not carried out):

(6) $$\text{WUE}=a{C_{a}}^{2}+bC_{a}+c$$

In equation (6), *C*_*a*_ is the atmospheric CO_2_ concentration, and *a*, *b* and *c* are the model evaluation coefficients (*a* and *b* have the unit of mol μmol^−1^, and the unit of *c* is (μmol CO_2_ mmol H_2_O^−1^)).

#### 2.3.4 Evaluation of adaptability of plants to elevated CO_2_ at different temperatures

In order to evaluate the adaptability of plants to elevated CO_2_, the photosynthetic parameter value corresponding to the 6th CO_2_ concentration (low, 50 μmol mol^−1^) was subtracted from the photosynthetic parameter value corresponding to the 7th CO_2_ concentration (environmental, 400 μmol mol^−1^), and the ratio between the difference of physiological parameters between CO_2_ concentration 7th and 6th and the maximum physiological parameters in the fitting curve was taken as the index of CO_2_ adaptation (*I*_*ac*_):

(7) $$I_{ac}=\frac{{\Delta P_{n}}_{\left(7-6\right)}}{A_{\max}}$$

where *I*_*ac*_ is CO_2_ adaptation index, ${\Delta P_{n}}_{\left(7-6\right)}$ is the difference of *P*_n_ betweens 7th and 6th CO_2_ concentration, *A*_max_ is the maximum value of *P*_n_, and the *I*_*ac*_ calculation method of *T*_r_ and WUE is the same as in equation (7), and the meanings of *P*_n_, *T*_r_ and WUE are the same as in equation (1).

In order to evaluate the continuous adaptability of plants to CO_2_ when CO_2_ is elevated and maintained at a certain level, the photosynthetic parameter value corresponding to the 7th CO_2_ concentration (400 μmol mol^−1^) was subtracted from the photosynthetic parameter value corresponding to the 8th CO_2_ concentration (400 μmol mol^−1^), and the ratio between the difference of physiological parameters between CO_2_ concentration 8th and 7th and the maximum physiological parameters in the fitting curve was taken as the index of CO_2_ secondary adaptation (*I*_*ac*II_):

(8) $$I_{ac\text{II}}=\frac{{\Delta P_{n}}_{\left(8-7\right)}}{A_{\max}}$$

where *I*_*ac*II_ is the CO_2_ quadratic adaptation index, ${\Delta P_{n}}_{\left(8-7\right)}$ is the difference of *P*_n_ between CO_2_ concentration 8th and 7th, *A*_max_ is the maximum value of *P*_n_, and the calculation method of *I*_*ac*II_ for *T*_r_ and WUE is the same as in equation (8), and the meanings of *P*_n_, *T*_r_ and WUE are the same as in equation (1).

The responses of photosynthetic parameters *P*_n_, *T*_r_ and WUE to temperature were estimated referring to equation (1) and the method of linear fitting.

#### 2.3.5 Data statistics and analysis

Sigmaplot 14.0 and MATLAB R2018a were used to creat plots and analyze. The normality of data sets was tested via Shapiro-Wilk test.

## 3 Results

### 3.1 Response characteristics of *P*_n_ to CO_2_ and temperature

**Fig. 3:**
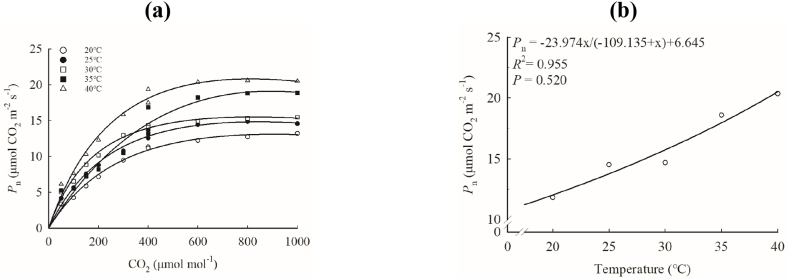
Response of *P*_n_ to CO_2_ (a) and *A*_max_ to temperature (b) in ‘Zhengdan 958’.

Under different temperatures, *P*_n_ of ‘Zhengdan 958’ first increased significantly with the increase of CO_2_ concentration, and then tended to be flat (Fig. 3a). With the increase of temperature, *A*_max_ increased continuously, ranging from 11.823 to 20.372 μmol m^−2^ s^−1^ (Table 1), and the growth rate gradually increased (Fig. 3b), *R*^2^ = 0.955. The values of CCP was extremely low, registering at approximately 10^−9^ μmol mol^−1^. The range of CSP was 399–542 μmol mol^−1^, and its response to temperature was smaller than *A*_max_, which further implied that CO_2_ doubling would not significantly improve *P*_n_ of maize.

**Table 1:** *P*_n_-CO_2_ response equations and maximum net photosynthetic rate, CO_2_ compensation point, photorespiration rate, and CO_2_ saturation point under different temperatures.

T (°C)	0–1000 μmol mol^−1^ CO_2_*P*_n_ fitting equations	*R*^*2*^	*A*_max_ (μmol m^−2^ s^−1^)	CCP (10^−9^ μmol mol^−1^)	CSP (μmol mol^−1^)
20.0	$y=0.057\left(1-2.832\times 10^{-4}x\right)x/\left(1+0.002x\right)-1.120\times 10^{-10}$	0.988	11.823	1.965	416.690
25.0	$y=0.074\left(1-2.822\times 10^{-4}x\right)x/\left(1+0.003x\right)-7.823\times 10^{-11}$	0.985	14.520	1.057	449.824
30.0	$y=0.101\left(1-2.296\times 10^{-4}x\right)x/\left(1+0.004x\right)-1.146\times 10^{-10}$	0.988	14.692	1.135	399.225
35.0	$y=0.061\left(1-3.772\times 10^{-4}x\right)x/\left(1+0.001x\right)-2.798\times 10^{-10}$	0.944	18.599	4.587	541.850
40.0	$y=0.102\left(1-3.099\times 10^{-4}x\right)x/\left(1+0.002x\right)-1.234\times 10^{-10}$	0.980	20.372	1.210	443.977

*R*^*2*^ is the determining coefficient; *A*_max_ is the maximum net photosynthetic rate; CCP is the CO_2_ compensation point; CSP is the CO_2_ saturation point. CCP in the table is calculated by method 1, and *A*_max_ and CSP are calculated by method 2.

### 3.2 Response characteristics of *T*_r_ to CO_2_ and temperature

**Fig. 4:**
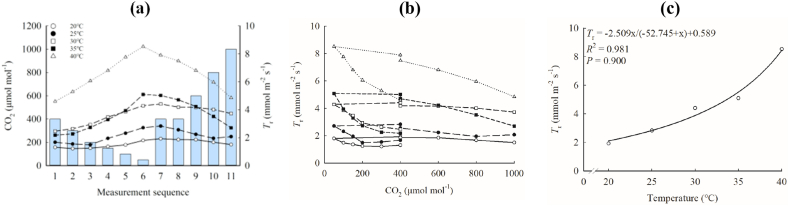
Response of *T*_r_ to CO_2_ (a, b) and *T*_rmax_ to temperature (c) in ‘Zhengdan 958’. In Fig. 4a, the gray bar graph represents the applied CO_2_ concentration, and the symbols of open and closed circles, open and closed squares, and open triangles represent 20°C, 25°C, 30°C, 35°C and 40°C in order.

**Fig. 5:**
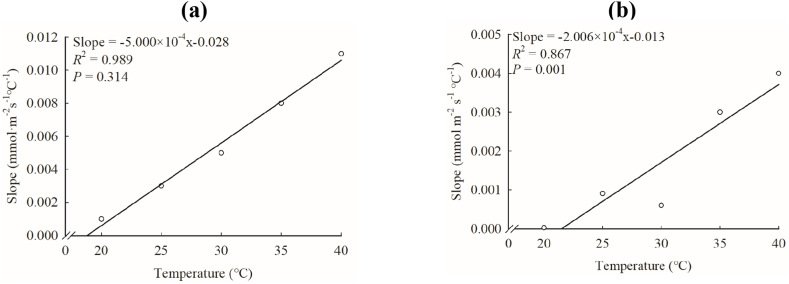
Response of the slope of CO_2_ decreasing section (a) and CO_2_ increasing section (b) to temperature in *T*_r_ of ‘Zhengdan 958’.

Regardless of whether the CO_2_ concentration was increasing or decreasing, there was a negative correlation between the *T*_r_ of maize and the ambient CO_2_ concentration. Furthermore, this phenomenon becomes more pronounced with increasing temperatures, resulting in a corresponding increase in *T*_*r*max_. (Fig. 4a). When the CO_2_ concentration gradually decreased from 400 μmol mol^−1^ to 50 μmol mol^−1^and then rose to 400 μmol mol^−1^ (CO_2_ concentration in the gray column in Fig. 4a), the *T*_r_ of the same CO_2_ concentration increased significantly (Fig. 4b). There was obvious hysteresis [11], which is a phenomenon of inconsistent values of photosynthetic parameters when photosynthetically relevant independent variables such as CO_2_ or light intensity are raised and lowered, in the response of *T*_r_ to ambient CO_2_ concentration. Moreover, the decreasing rate of *T*_rmax_ during the gradual decrease of CO_2_ concentration from 400 to 50 μmol mol^−1^ (slope range: 0.001–0.011) was higher than the increasing rate of *T*_rmax_ during the gradual increase of CO_2_ concentration from 50 to 1000 μmol mol^−1^ (slope range: 0.308 × 10^−4^–0.004) (Table 2). The slope of CO_2_ concentration decreasing and increasing increases linearly with the increase of temperature, with *R*^2^ of 0.989 and 0.967, respectively, both reaching significant levels (Fig. 5a and b). When the CO_2_ concentration gradually increased from 400 μmol mol^−1^ to 800 μmol mol^−1^, *T*_r_ decreased significantly, and the decrease was about 30 % of the decreasing CO_2_ concentration. The response of *T*_rmax_ to temperature increased with the increase of temperature, and the highest was 8.526 mmol m^−2^ s^−1^. The relationship of *T*_rmax_ to temperature could be fitted by right-angle hyperbola (Fig. 4c), and *R*^2^ was 0.981, which was extremely significant.

**Table 2:** *T*_r max_ and its piecewise linear fitting equations at different temperatures.

T (°C)	*T*_rmax_ (mmol m^−2^ s^−1^)	The CO_2_ concentration of *T*_rmax_ (μmol mol^−1^)	Fitted segments	linear fitting equation	*R*^2^	The ratio of latter slope between initial slope
20.0	1.922	400	Initial Latter	y = −0.001x+1.644 y = −0.308 × 10^−4^x+1.952	0.535 0.545	0.031
25.0	2.827	400	Initial Latter	y = −0.003x+2.526 y = 9.081 × 10^−4^x+2.883	0.617 0.726	0.303
30.0	4.405	400	Initial Latter	y = −0.005x+4.325 y = −6.004 × 10^−4^x+4.453	0.908 0.718	0.120
35.0	5.092	50	Initial Latter	y = −0.008x+4.799 y = −0.003x+6.148	0.903 0.987	0.375
40.0	8.526	50	Initial Latter	y = −0.011x+8.739 y = −0.004x+9.057	0.953 0.958	0.364

CO_2_ concentration gradually decreasing part：400–50 μmol mol^−1^;CO_2_ concentration gradually increasing part：50–1000 μmol mol^−1^.

### 3.3 Response characteristics of WUE to CO_2_ and temperature

**Fig. 6:**
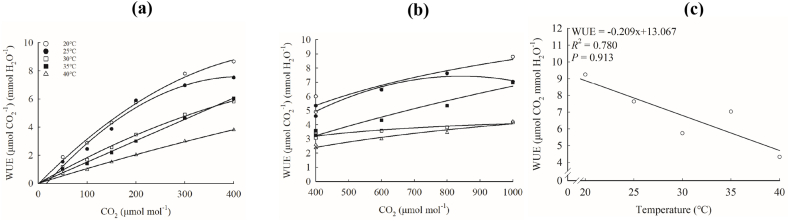
Response of WUE to CO_2_ (a: 0–400 μmol mol^−1^; b:400–1000 μmol mol^−1^) and WUE_max_ to temperature (c) in‘Zhengdan 958’.

The WUE of ‘Zhengdan 958’ decreased with the increase of temperature and increased with the increase of CO_2_ concentration (Fig. 6a and b), but the slope of WUE fitting equation in the range of 0–400 μmol mol^−1^ was higher than that in the range of 400–1000 μmol mol^−1^ (Table 3), that is, after CSP, *P*_n_ no longer increased, and *T*_r_ still decreased due to the decrease of *G*_s_, but the change rate decreased.

**Table 3:** 0–400 μmol mol^−1^ and 400–1000 μmol mol^−1^ CO_2_ WUE fitting equations and WUE_max_ in corresponding intervals at different temperatures.

Fitting equation corresponding interval	T (°C)	WUE fitting equations	*R*^*2*^	WUE_max_ (μmol CO_2_ mmol H_2_O^−1^)
0–400 μmol mol^−1^	20.0	$y=-3.278\times 10^{-5}x^{2}+0.035x-0.044$	0.993	8.711
	25.0	$y=-4.820\times 10^{-5}x^{2}+0.040x-0.656$	0.978	7.632
	30.0	$y=-9.905\times 10^{-6}x^{2}+0.018x+0.125$	0.994	5.740
	35.0	$y=-5.169\times 10^{-16}x^{2}+0.015x+0.075$	0.995	6.075
	40.0	$y=-7.759\times 10^{-16}x^{2}+0.009x+0.175$	0.996	3.775
400–1000 μmol mol^−1^	20.0	$y=-1.159\times 10^{-16}x^{2}+0.006x+3.255$	0.933	9.255
	25.0	$y=-1.271\times 10^{-5}x^{2}+0.021x-1.583$	0.939	7.091
	30.0	$y=-2.314\times 10^{-7}x^{2}+0.002x+2.519$	0.928	4.288
	35.0	$y=-1.426\times 10^{-16}x^{2}+0.006x+1.031$	0.976	7.031
	40.0	$y=-1.032\times 10^{-16}x^{2}+0.003x+1.331$	0.978	4.331

**Fig. 7:**
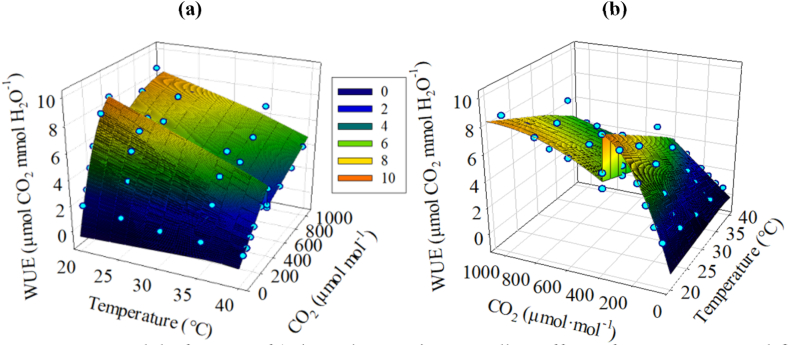
Response model of WUE of ‘Zhengdan 958’ to coupling effect of temperature and CO_2_. (a: temperature response perspective; b:CO_2_ response perspective)

WUE_max_ decreased approximately linearly with the increase of temperature (*R*^2^ = 0.780) (Fig. 6c). The piecewise fitting results of the WUE-CO_2_ response curve (Table 3) show that the WUE_max_ in the initial stages was higher than the corresponding value in the latter stages at 20 °C, 25 °C and 30 °C, but the comparison of WUE_max_ in the initial stages and the latter stages was just the opposite at 35 °C and 40 °C, and the comparison of WUE_max_ in the initial stages and the latter stages from 20 °C to 40 °C implied that the response hysteresis of WUE to CO_2_ was weakened under high temperature. In this study, WUE_max_ was obtained under relatively low temperature (20 °C). The 3D response model of maize WUE to the coupling effect of temperature and CO_2_ (Fig. 7a and b) could clearly show that appropriate low temperature and high CO_2_ concentration can improve WUE. Nevertheless, due to the increase of global CO_2_ concentration, the temperature will increase, and the planting area of maize needs to be further moved northward to achieve high WUE. However, whether the increase of WUE under the conditions of relatively low temperature and high CO_2_ concentration will help to lift the restriction of relatively less precipitation on maize growth in the north needs further study.

### 3.4 Adaptability evaluation of maize *P*_n_, *T*_r_ and WUE to elevated CO_2_ at different temperatures

**Fig. 8:**
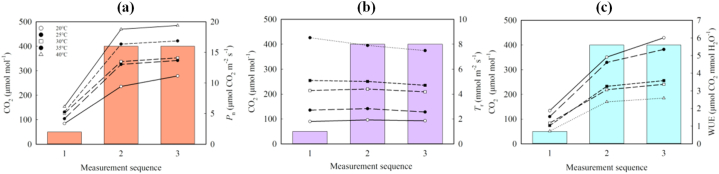
Adaptive response of *P*_n_ (a), *T*_r_ (b) and WUE (c) of ‘Zhengdan 958’ to CO_2_. In Fig. 8a,b,c, the gray bar graph represents the applied CO_2_ concentration, and the symbols of open and closed circles, open and closed squares, and open triangles represent 20°C, 25°C, 30°C, 35°C and 40°C in order.

The adaptability of *P*_n_, *T*_r_ and WUE of ‘Zhengdan 958’ to elevated CO_2_ was evidently different in response to temperature: at 25 °C–40 °C, the level of *I*_*ac*_ of *P*_n_ to the sudden increase of CO_2_ from low concentration (50 μmol mol^−1^) to environmental concentration (400 μmol mol^−1^) was high; when the temperature was low (20 °C ∼ 30 °C), the *I*_*ac*_ value of *T*_r_ was positive, but it was negative when the temperature was high (35 °C ∼ 40 °C). The final *I*_*ac*_ of WUE (*P*_n_/*T*_r_) was the highest at 25 °C and 40 °C (Fig. 8a,b,c, Table 4), which showed that maize WUE has good adaptability to low CO_2_ concentration rising to ambient CO_2_ concentration at the corresponding temperature. Furthermore, in this process, the better the adaptability it is, the weaker the hysteresis phenomenon. In this study, the extreme values of *I*_*ac*_ of *P*_n_, *T*_r_ and WUE (0.621, −0.073 and 0.405) were the highest at 40 °C, and the hysteresis of WUE was the weakest.

**Table 4:** $I_{ac}$ and $I_{acII}$ of *P*_n_, *T*_r_ and WUE at different temperatures.

T (°C)	*P*_n_ (μmol m^−2^ s^−1^)			*T*_r_ (mmol m^−2^ s^−1^)			WUE (μmol CO_2_ mmol H_2_O^−1^)		
	$I_{ac}$	$I_{ac\text{II}}$	$I_{ac}$ +$I_{ac\text{II}}$	$I_{ac}$	$I_{ac\text{II}}$	$I_{ac}$ +$I_{ac\text{II}}$	$I_{ac}$	$I_{ac\text{II}}$	$I_{ac}$ +$I_{ac\text{II}}$
20.0	0.514	0.145	0.659	0.065	−0.034	0.031	0.348	0.125	0.473
25.0	0.611	0.044	0.655	0.043	−0.095	−0.052	0.403	0.096	0.499
30.0	0.576	0.042	0.618	0.027	−0.051	−0.024	0.316	0.052	0.368
35.0	0.597	0.028	0.625	−0.014	−0.061	−0.075	0.317	0.046	0.363
40.0	0.621	0.030	0.651	−0.073	−0.048	−0.121	0.405	0.052	0.457

As for the continuous adaptation of maize to CO_2_ of maize, the *I*_*ac*II_ of 20 °C *P*_n_, 25 °C *T*_r_, 20 °C and 25 °C WUE were significantly higher than *I*_*ac*II_ of other corresponding temperature parameters, among which *I*_*ac*II_ of WUE was higher at 20 °C than at 25 °C, indicating that relatively low temperature is beneficial to the continuous adaptation of maize WUE under ambient CO_2_ concentration.

For the continuous response (*I*_*ac*_ + *I*_*ac*II_), except for the weak response at 30 °C and 35 °C, the continuous adaptation level of *P*_n_ at other temperatures was almost similar; the negativity of *T*_r_'s continuous adaptability gradually increases with temperature. The continuous adaptability of WUE reached a high level at 20 °C, 25 °C and 40 °C, and *I*_*ac*_ + *I*_*ac*II_ at 25 °C was the highest, reaching 0.499. This may indicate that both room temperature (25 °C) and high temperature (40 °C) can effectively reduce the hysteresis effect of WUE for a long time, but the mechanism may be different.

## 4 Discussion

The analysis of the response curve of ‘Zhengdan 958’*P*_n_ to CO_2_ and temperature shows that *P*_n_ increased rapidly at first and then tended to saturation with the increase of CO_2_ (Fig. 3a), which may be related to the limitation of triose phosphate utilization (TPU) [12]. Moreover, its CSP was between 399 and 542 μmol mol^−1^, indicating that the increase of CO_2_ based on the existing ambient CO_2_ concentration will not significantly increase its photosynthesis. However, the *P*_n_ increased significantly with temperature during test temperature regime (Fig. 3b), which is different from the cognition that high temperature inhibits the activity of photosynthesis-related enzymes. This observation suggests that the *P*_n_-T response of maize may have a higher optimal temperature under field conditions. Ghannoum et al. [13] thought that the *C*_i_ of C4 plants was much larger than that of *C*_*a*_, but the results of this study show that the *C*_i_ value of ‘Zhengdan958’ was slightly lower than that of *C*_*a*_, and the change trend of *C*_i_ and *C*_*a*_ was highly similar at 20 °C ∼ 30 °C, which suggests that the increase of *C*_*a*_ will improve *P*_n_ of C4 plants, which is further supported by the fact that *I*_*ac*_ of *P*_n_ was not less than 0.514 at different temperatures under the sudden change of *C*_*a*_. However, it is worth noting that under the conditions of 35 °C and 40 °C and low CO_2_ (50–400 μmol mol^−1^), *C*_i_ hardly responded to *C*_*a*_ (Fig. S1a), which indicates that higher temperature and lower CO_2_ concentration will inhibit the response level of C4 plant *P*_n_ to the increase of *C*_*a*_. At different temperatures, CCP tended to 0 (Table 1). This is because C4 plants rely on the Kranz structure to prevent CO_2_ escaping from bundle sheath cells, which weakens photorespiration, and CSP was also extremely small. Though CSP was approximately linear due to the obvious decrease of 30 °C value, its growth trend was similar to that of *A*_max_, which is consistent with the known theory.

For the estimation of photosynthetic parameters, compared with the analytical solution of the model (Method 1), the linear fitting estimation (Method 2) is more accurate in estimating *A*_max_ and CSP (Table 1, Table 5). However, for C4 plants with weak photorespiration such as maize, method 2 is not suitable for fitting their CCP.

**Table 5:** Maximum net photosynthetic rate, CO_2_ compensation point, photorespiration rate, and CO_2_ saturation point of ‘Zhengdan 958'under different temperatures (Method 1).

T (°C)	*A*_max_ (μmol m^−2^ s^−1^)	CSP (μmol mol^−1^)	*A*_max_ measured value (μmol m^−2^ s^−1^)
20.0	13.654	919.696	≈13
25.0	13.481	803.463	≈14.5
30.0	15.707	823.010	≈15
35.0	19.087	910.789	≈18.5
40.0	23.655	865.073	≈20

At different temperatures, the *T*_r_ of ‘Zhengdan 958’ shows the characteristics of decreasing with the increase of CO_2_ concentration (Fig. 4a and b), because the increase of CO_2_ leads to the decrease of *G*_s_, which leads to the decrease of transpiration (Fig. S1b). At the same time, the fitting result of *T*_r_-T curve (Fig. 4c) shows that *T*_r_ increased continuously with the increase of temperature, which was consistent with the observation of Mott and Peak [14], which may be due to the fact that the temperature rise increases the steam pressure difference between the inside and outside of the blade, which leads to the increase of *T*_r_. However, because *T*_r_ is affected by stomatal resistance, its response to temperature is complicated. In the existing research, the response modes of *T*_r_-T were not consistent [15], but the hysteresis phenomenon in this study and the difference of *I*_*ac*_'s response to temperature also show that the clear mechanism of *T*_r_-T response still needs further study and discussion. It's worth mentioning that the hysteresis phenomenon may be caused by the joint influence of *G*_s_ and steam pressure inside and outside the leaf. However, this hysteresis phenomenon does not appear in photosynthesis, which may be because transpiration is affected by stomatal opening for a short time, while the photosynthetic rate is also influenced by non-stomatal factors which play a major role. Thus, transpiration is more affected by changes in stomatal conductance than photosynthesis, and finally the hysteresis phenomenon in transpiration does not appear in photosynthesis. However, according to Papanatsiou et al. [16], the relevant study of stomatal dynamics may effectively separate the effects of CO_2_ assimilation and water loss temporarily, so further work related to stomatal dynamics is needed.

In the study, the change of *T*_r_ with CO_2_ concentration showed clear hysteresis as evident by the fact that when the low CO_2_ concentration (50 μmol mol^−1^) rose back to the ambient CO_2_ concentration (400 μmol mol^−1^), the *T*_r_ at the same CO_2_ concentration increased. However, when the CO_2_ concentration gradually increased from 400 μmol mol^−1^ to 800 μmol mol^−1^, *T*_r_ decreased significantly, and the decrease rate was about 30 % of the decreasing period of CO_2_ concentration. The ratio of the decrease rate of the initial and latter stages can be used to describe the hysteresis phenomenon in transpiration. The appearance of hysteresis phenomenon is probably due to the stomatal opening caused by low CO_2_ concentration treatment in the early stage, which leads to the failure of *T*_r_ to recover in time after sufficient CO_2_ supply is restored. However, whether this hysteresis phenomenon is universal and its sensitivity and degree affected by environmental factors need further study.

In this study, the photosynthetic CSP of ‘Zhengdan 958’ is close to the ambient CO_2_ concentration (the highest was 542 μmol mol^−1^) at different temperatures, which is the result of long-term adaptation to the environment. However, when CO_2_ increased, *G*_s_ was decreased (Fig. S1b), and *T*_r_ was further inhibited, and the inhibition degree of CO_2_ increased on *T*_r_ was lower than that of temperature increased on *T*_r_. This shows that the effect of elevated ambient CO_2_ concentration on transpiration and photosynthesis of plants is mainly realized by its temperature rise, while the direct effect of CO_2_ is weaker than that of temperature.

In this study, at relatively low temperature (20 °C ∼ 30 °C), the WUE_max_ in the initial stages was higher than the corresponding value of the latter stages, but the comparison of WUE_max_ in the initial stages and the latter stages was just the opposite under high temperature (35 °C and 40 °C). This shows that WUE's long-term sustainable adaptability to the increase of CO_2_ concentration is weak at relatively low temperature. However, the short-term adaptability (*I*_*ac*_) reached a high level at 25 °C and 40 °C respectively. It is worth noting that CO_2_ concentration was set from 50 μmol mol^−1^ to 400 μmol mol^−1^ in this study, which can assess the adaptability of the plants to elevated CO_2_. However, the relevant parameters measured were all instantaneous indicators, it may not reflect the adaptability of plants to the long-term elevated CO_2_, so further research is still needed.

The maximum value of WUE appeared at 1000 μmol mol^−1^ CO_2_ and 20 °C, which shows that relatively low temperature and high CO_2_ are effective conditions to achieve high WUE. The research of Li et al. [17] also showed that when the temperature increased above 1.5 °C, the “fertilizer effect” of *C*_*a*_ increase will become uncertain; and if the temperature continued to increase, the “fertilizer effect” may disappear completely, which is well reflected in the WUE_max_-T response (Fig. 5c) and the coupling model (Fig. 6a) in this study. At different temperatures, the increase of CO_2_ concentration significantly increased the WUE of maize (Fig. 5a and b), which is the same as the conclusion of Sreeharsha et al. [18] which state that the increase of atmospheric CO_2_ concentration can increase the WUE of crops. The increase of WUE with the increase of CO_2_ concentration is the result of CO_2_ increasing promoting photosynthesis and inhibiting transpiration. According to Liu et al. [19], the drivers behind the increase of WUE differ depending on whether temperatures are relatively low or high. In the case of relatively low temperatures, the rise in WUE is primarily attributable to an elevation in CO_2_ concentration, coupled with a decline in *G*_s_, which suppresses leaf transpiration. Conversely, in high temperature conditions, the increase of CO_2_ levels mitigates the detrimental impact of heat stress on photosynthetic organs, thereby preserving elevated levels of WUE.

In addition to ‘Zhengdan 958’, this study also measured and fitted the photosynthetic parameters of ‘Jinkai 2’, another major maize variety in Guanzhong Region, Shaanxi Province, China, in reponse to CO_2_. The values of *P*_n_, *T*_r_ and WUE of ‘Zhengdan 958’ were different from those of ‘Jinkai 2’, but the response trend to CO_2_ was similar (Fig. S2**a,b,c**), which may indicate that the variety difference has influence on the CO_2_ response level of maize WUE related parameters, but has little influence on the response trend.

Therefore, the photosynthesis and transpiration of maize at ear stage have obvious coupling responses to temperature and CO_2_. The response model of WUE to the coupling effects of temperature and CO_2_ is established by analyzing the characteristics of *P*_n_ and *T*_r_ changes corresponding to different temperatures and CO_2_, which has important reference value for improving the yield potential of crops under global warming and predicting the future crop yield changes.

To sum up, at the same temperature, the *P*_n_ and WUE of maize of CO_2_ concentration positively impacted the increase, whereas *T*_r_ exhibited a negative correlation with CO_2_ concentration. In the same CO_2_ concentration, *P*_n_ and *T*_r_ were positively correlated with temperature, while WUE decreased with the increase of temperature. The corresponding environmental conditions of WUE_max_ were 1000 μmol mol^−1^ CO_2_ and 20 °C. The short-term adaptation level of *P*_n_ to sudden change of CO_2_ is relatively consistent, but the short-term adaptation ability of *T*_r_ is quite different, which finally shows that the short-term adaptation ability of WUE reached a high level at relatively low temperature (20 °C and 25 °C) and high temperature (40 °C) respectively. The responses of *P*_n_, *T*_r_ and WUE in maize had little difference among varieties.

With the increase of global CO_2_ concentration, the temperature will further increase, but the doubling of CO_2_ will not further increase *P*_n_ of maize. However, the increase of temperature will aggravate transpiration, which will lead to the decrease of WUE. This, in turn, poses more significant challenges to global food security. The planting areas of maize will move northward due to global warming, which will further aggravate the current situation of water shortage in the north. Facing this challenge, it is also an important research direction to strengthen the research on agricultural management measures or cultivation system to improve maize WUE while breeding high WUE varieties through modern molecular biology technology.

## Data availability statement

Data associated with this study has never been deposited into a publicly available repository, and data will be made available on request.

## CRediT authorship contribution statement

**Sicong Sun:** Writing – review & editing, Writing – original draft, Methodology, Formal analysis, Data curation, Conceptualization. **Xinquan Hu:** Writing – review & editing, Methodology, Investigation, Conceptualization. **Yongsheng Wei:** Writing – review & editing, Supervision, Conceptualization. **Xiaoxiao Chen:** Investigation. **Yanzheng Li:** Investigation. **Jun Cao:** Investigation.

## Declaration of competing interest

The authors declare that they have no known competing financial interests or personal relationships that could have appeared to influence the work reported in this paper.
