# Supplementary material for: Response of WUE of maize at ear stage to the coupling effect of CO2 and temperature
Source: Heliyon. 2023 Dec 19;10(1):e23646. doi: 10.1016/j.heliyon.2023.e23646 (PMC10784164; doi:10.1016/j.heliyon.2023.e23646)

Table S1 Meanings and Units of Abbreviations

| Abbreviations | Meanings |
| --- | --- |
| *A*_max_ | Maximum net photosynthetic rate (μmol m^−2^ s^−1^) |
| *C_a_* | Atmospheric CO_2_ concentration (μmol mol^-1^) |
| CCP | CO_2_ compensation point |
| *C*_i_ | Intercellular CO_2_ concentration (μmol mol^-1^) |
| CSP | CO_2_ saturation point |
| *G*_s_ | Stomatal conductance (mmol m^-2^ s^-1^) |
| *I_a_*_c_ | The index of CO_2_ adaptation |
| *I_a_*_cII_ | The index of CO_2_ secondary adaptation |
| PAR | Photosynthetically Active Radiation (μmol m^−2^ s^−1^) |
| *P*_n_ | Net Photosynthate (μmol CO_2_ m^-2^ s^-1^) |
| *T*_r_ | Transpiration rate (mmol m^-2^ s^-1^) |
| WUE | Water Use Efficiency (μmol CO_2_ mmol H_2_O^-1^) |

| Table S2 Response of *C*_i_ and *G*_s_ of' Zhengdan 958' to *C_a_* at different temperatures | | | |
| --- | --- | --- | --- |
| T (℃) | *C_a_* (μmol mol^-1^) | *C*_i_ (μmol mol^-1^) | *G*_s_ (mmol m^-2^ s^-1^) |
| 20 | 400 | 265.620 | 0.182 |
|  | 300 | 183.904 | 0.164 |
|  | 200 | 115.808 | 0.167 |
|  | 150 | 87.118 | 0.187 |
|  | 100 | 58.537 | 0.211 |
|  | 50 | 42.903 | 0.272 |
|  | 400 | 323.524 | 0.294 |
|  | 400 | 305.058 | 0.287 |
|  | 600 | 492.924 | 0.285 |
|  | 800 | 666.347 | 0.254 |
|  | 1000 | 834.194 | 0.225 |
| 25 | 400 | 206.619 | 0.127 |
|  | 300 | 140.975 | 0.126 |
|  | 200 | 76.681 | 0.141 |
|  | 150 | 71.717 | 0.192 |
|  | 100 | 48.531 | 0.224 |
|  | 50 | 33.881 | 0.275 |
|  | 400 | 299.498 | 0.291 |
|  | 400 | 282.492 | 0.255 |
|  | 600 | 448.934 | 0.219 |
|  | 800 | 604.928 | 0.189 |
|  | 1000 | 725.180 | 0.141 |
| 30 | 400 | 199.117 | 0.137 |
|  | 300 | 131.424 | 0.149 |
|  | 200 | 82.232 | 0.171 |
|  | 150 | 65.015 | 0.212 |
|  | 100 | 43.344 | 0.285 |
|  | 50 | 34.478 | 0.245 |
|  | 400 | 293.005 | 0.294 |
|  | 400 | 283.991 | 0.278 |
|  | 600 | 467.055 | 0.263 |
|  | 800 | 652.485 | 0.249 |
|  | 1000 | 831.819 | 0.222 |
| 35 | 400 | 2.643 | 0.058 |
|  | 300 | -6.586 | 0.060 |
|  | 200 | 1.001 | 0.074 |
|  | 150 | 4.150 | 0.091 |
|  | 100 | 7.341 | 0.111 |
|  | 50 | 9.441 | 0.152 |
|  | 400 | 180.753 | 0.151 |
|  | 400 | 159.869 | 0.139 |
|  | 600 | 302.875 | 0.122 |
|  | 800 | 425.636 | 0.099 |
|  | 1000 | 510.077 | 0.074 |
| 40 | 400 | 20.724 | 0.085 |
|  | 300 | -0.546 | 0.099 |
|  | 200 | -2.208 | 0.116 |
|  | 150 | -0.456 | 0.133 |
|  | 100 | 3.072 | 0.155 |
|  | 50 | 4.051 | 0.174 |
|  | 400 | 151.442 | 0.159 |
|  | 400 | 132.405 | 0.149 |
|  | 600 | 277.798 | 0.132 |
|  | 800 | 416.869 | 0.113 |
|  | 1000 | 523.025 | 0.089 |

Fig. S1 Response of *C*_i_ (a) and *G*_s_ (b) of ‘Zhengdan 958’ to CO_2_ at different temperatures

In Fig. **S1a,b**, the gray bar graph represents the applied CO_2_ concentration, and the symbols of open and closed circles, open and closed squares, and open triangles represent 20℃, 25℃, 30℃, 35℃ and 40℃ in order.

**(a)**

**(b)**


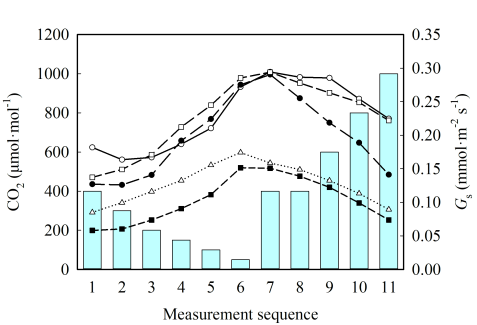

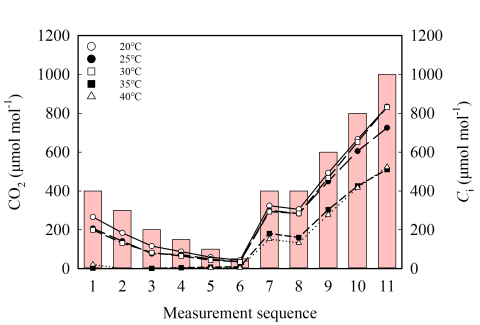


Fig. S2 Response of *P*_n_ (a), *T*_r_ (b) and WUE (c) of ‘Zhengdan 958’ and ‘Jinkai 2’ to CO_2_ at 30℃

In Fig. **S2a,b,c**, the gray bar graph represents the applied CO_2_ concentration.

**(a)**

**(b)**

**(c)**


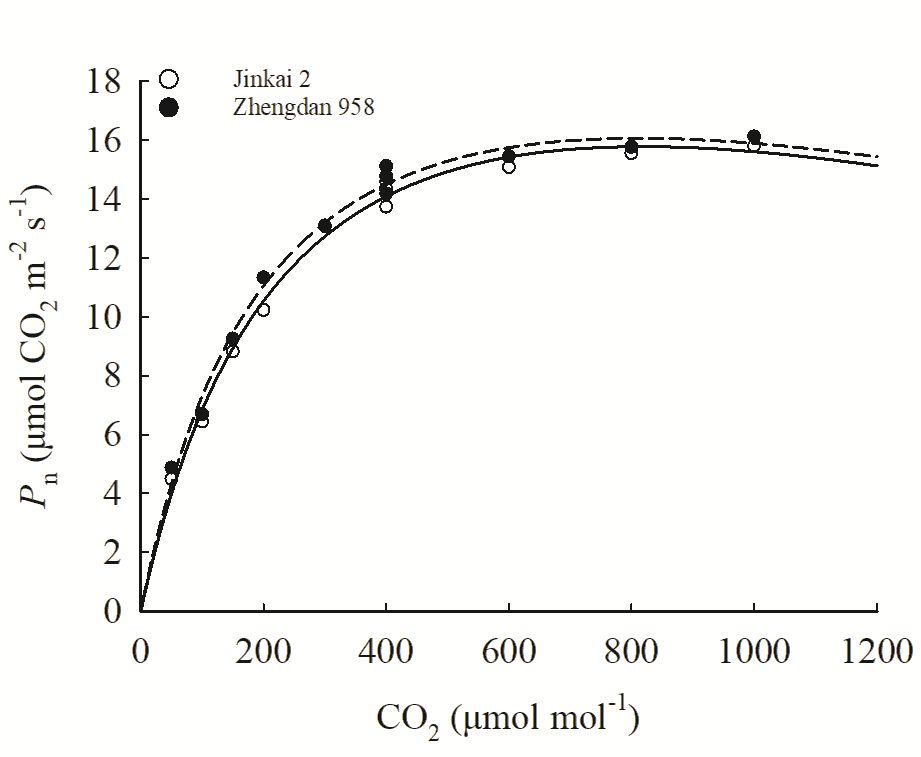

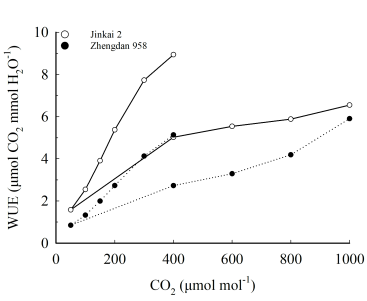

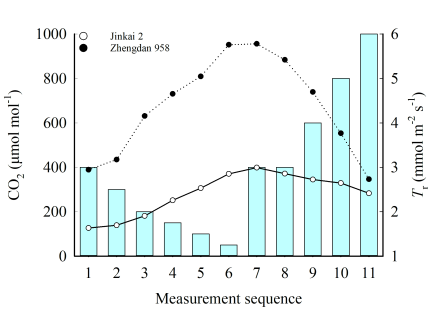

Supplement: Multimedia component 1 [file mmc1.docx]
